# Torsion of the Appendix Testis in a Neonate

**DOI:** 10.1155/2016/9183196

**Published:** 2016-06-09

**Authors:** Arvind Krishnan, Mark A. Rich, Hubert S. Swana

**Affiliations:** ^1^University of Central Florida College of Medicine, Orlando, FL 32827, USA; ^2^Nemours Children's Hospital, Orlando, FL 32827, USA

## Abstract

Torsion of the appendix testis is a rare cause of scrotal swelling in the neonatal period. We present a case of torsion of the appendix testis in a one-day-old male. We discuss the physical examination and radiologic studies used to make the diagnosis. Nonoperative therapy was recommended and the patient has done well. Recognition of this condition in the neonatal period can prevent surgical intervention and its associated risks.

## 1. Introduction

There are several causes of scrotal swelling in the neonate. The most common causes include testicular torsion, neoplasms, supernumerary testis, splenogonadal fusion, and adrenal rests [1, 2]. Torsion of the appendix testis in the neonatal period is exceedingly rare. We discuss the diagnosis and management of appendix testis torsion in a neonate and review the literature.

## 2. Case Presentation

**Figure 1:**
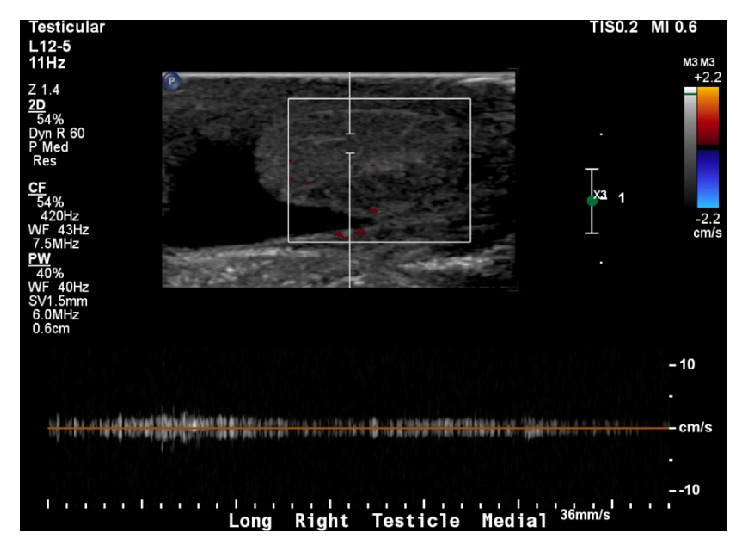
Doppler image of right testis. Normal testicular morphology was noted and no intratesticular masses were identified. Normal blood flow to the testicle was demonstrated.

**Figure 2:**
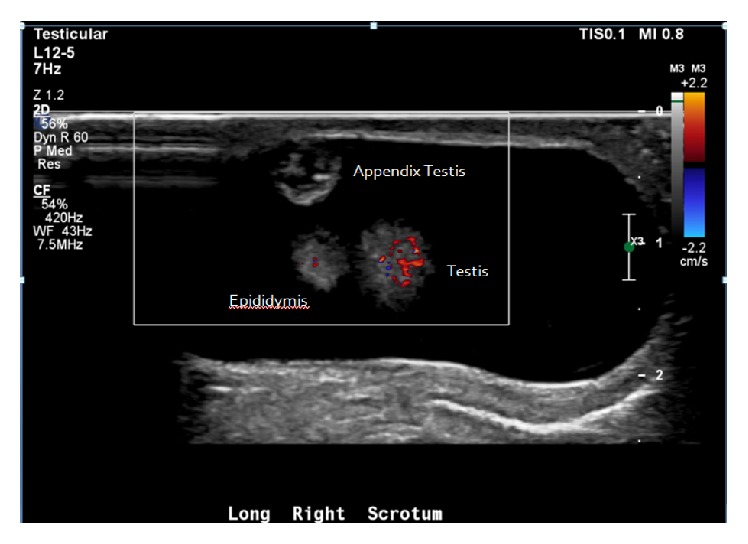
Doppler ultrasonography revealed no blood flow to the appendix testis. Normal blood flow to the testis and epididymis were demonstrated. Right hydrocele was also identified.

**Figure 3:**
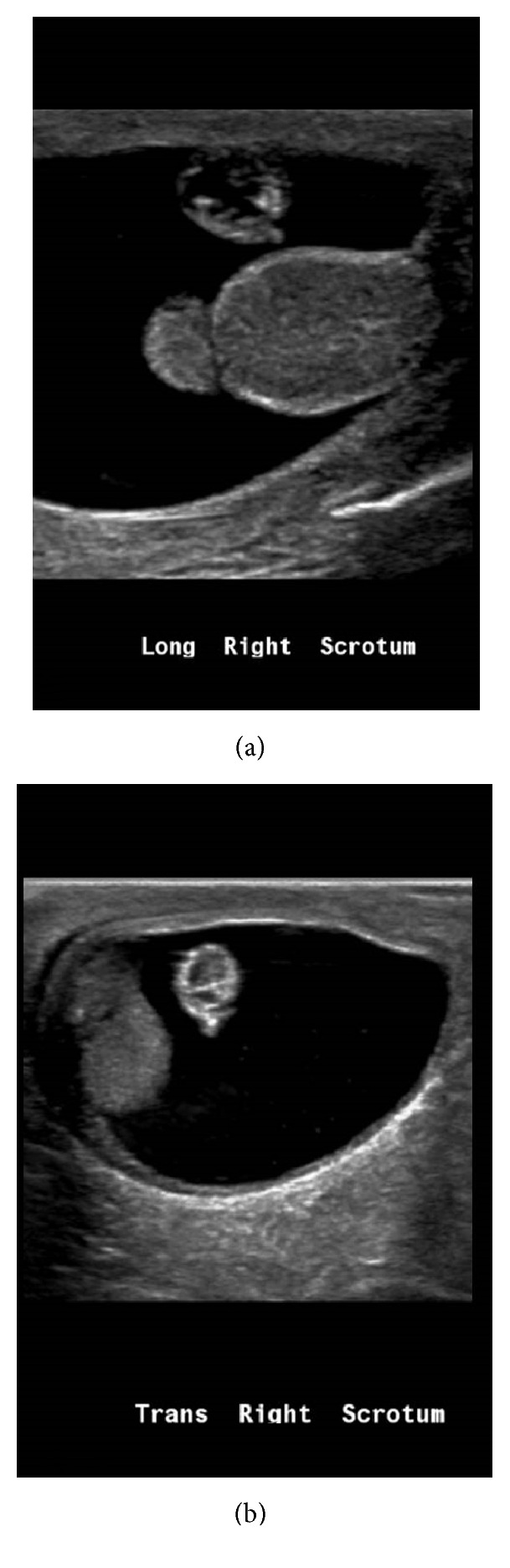
(a) Initial scrotal ultrasound showing large avascular extratesticular mass measuring 0.5 × 0.55 × 0.57 cm. (b) Six-week follow-up scrotal ultrasound shows decrease in size of extratesticular mass measuring 0.39 × 0.37 × 0.44 cm.

A one-day-old newborn was found on initial exam to have right sided scrotal swelling and urologic consultation was obtained. The patient was the product of a 39-week gestation born via an uncomplicated vaginal delivery. The pregnancy was notable only for well-controlled maternal diabetes. On examination, the patient was well appearing. His abdomen was soft and without masses. No swelling was appreciated in his groins. He had had a bluish discoloration of the right hemiscrotal skin. Scrotal ultrasonography demonstrated normal testicular morphology and good testicular blood flow. Large right hydrocele was identified but no testicular masses were noted (Figure 1). Adjacent to the right testicle, an avascular extratesticular mass was noted, measuring approximately 6 mm (Figure 2). Based on the ultrasound and physical exam findings, the diagnosis of torsion of the appendix testis was considered. Clinically, the patient was stable and emergent causes of scrotal swelling were excluded. Due to the fact that the patient was stable and was not experiencing discomfort or urinary tract symptoms, close observation and local care were recommended. Empiric antibiotics were not administered as no clinical or radiologic signs of epididymitis were present. Ultrasonography showed an epididymis with normal morphology and without hypervascularity and no erythema of the scrotum was noted on physical examination. The patient was discharged after routine hospitalization and returned 6 weeks later for follow-up. He was well appearing and asymptomatic. Repeat ultrasonography demonstrated that the previously identified extratesticular mass and hydrocele had decreased significantly in size (Figure 3).

## 3. Discussion

The appendix testis is a vestige of the Müllerian duct that remains as a nonfunctional remnant during male embryological development [3]. The morphology can vary from a small nodule to a longer protuberance. Torsion can occur in longer pedunculated appendices, compromising the blood supply. Acutely they cause local inflammation and pain. Infarction ultimately leads to atrophy and resolution of symptoms. It most often presents in 7–14-year-old boys with acute scrotal pain with swelling in the anterosuperior region of the testicle [4, 5]. There is typically a pathognomonic “blue dot” sign, which represents the swollen appendix testis within the scrotal sac that has a cyanotic hue. It may be palpated as a 2-3 mm firm nodule in the upper pole of the testicle. Patients can present with pain but usually do not experience systemic signs of fever, nausea, or vomiting. Most pediatric patients with torsion of the appendix testis can be treated nonoperatively with pain control and rest. Occasionally the pain persists and surgery may be required to expedite the recovery process [6].

Etiologies of scrotal swelling in the neonate are categorized as intratesticular or extratesticular. Intratesticular causes include testicular torsion, neoplasm, and supernumerary testis, whereas extratesticular causes include hematoma, hydrocele, inguinal hernia, and extension of systemic disease [7]. Torsion of the appendix testis in neonates is extremely rare. Only a handful of reports have been published in primary literature. In 1969, Chiles and Foster Jr. reported a case of a 16-hour-old male who was originally thought to have testicular torsion and treated with scrotal exploration. At surgery it was discovered that the patient had torsion of the appendix testis and the cord itself was normal [8]. Due to the rarity of torsion of the appendix testis in neonates, management has not been standardized. The European Association of Urology (EAU) guidelines for acute scrotum in neonates recommend surgical intervention [9]. If emergent causes are suspected, such as incarcerated hernia or testicular torsion, then surgical exploration is warranted. In our case, emergent causes of scrotal swelling were excluded.

Torsion of the appendix testis is an extremely rare cause of scrotal swelling in neonates. Ultrasonography is an important diagnostic tool in the diagnosis of a neonate with scrotal swelling. If one suspects appendix testis torsion and emergent causes of scrotal swelling are ruled out, conservative management can be considered.

## 4. Conclusions

Torsion of the appendix testis is a rare cause of scrotal swelling in neonates. Accurate diagnosis is important as the treatment is nonoperative. Ultrasonography is an important diagnostic tool and if one is confident about the diagnosis, conservative management can be considered.
